# Management of Locally Advanced Cutaneous Squamous Cell Carcinoma of the Scalp: A Case Report and Literature Review

**DOI:** 10.7759/cureus.34938

**Published:** 2023-02-13

**Authors:** Elle Nuttall, Ryan M Hudnall, Tony Richa

**Affiliations:** 1 Otolaryngology - Head and Neck Surgery, Creighton University School of Medicine, Omaha, USA; 2 Otolaryngology - Head and Neck Surgery, University of Nebraska Medical Center, Omaha, USA

**Keywords:** cutaneous squamous cell carcinoma, neurosurgery, head and neck cancer surgery, head and neck surgery, cutaneous malignancy, head and neck tumors and diseases, head and neck neoplasms, squamous cell carcinoma

## Abstract

Cutaneous squamous cell carcinoma (cSCC) is a common malignancy in the head and neck region. Although rarely metastatic, it has the potential to become locally aggressive and invade surrounding structures. The involvement of the scalp with cSCC presents a unique surgical challenge given its proximity to vital structures. We present a case of locally advanced cutaneous scalp squamous cell carcinoma with involvement of the skull and dural involvement not previously demonstrated on imaging. This case required complex multidisciplinary surgical management from both neurosurgery and head and neck surgery for treatment, preservation of essential tissues, and cosmesis.

## Introduction

Cutaneous squamous cell carcinoma (cSCC) represents 20-50% of all skin cancers [1-3]. This cancer most commonly affects the head and neck region due to its high exposure to ultraviolet (UV) radiation [2,4]. We present a case of a locally advanced scalp cSCC with involvement of the calvarium and dural involvement not previously demonstrated on imaging. This case posed a unique surgical challenge given the size of the defect after extensive local excision and involvement of the deep cranial structures.

## Case presentation

**Figure 1:**
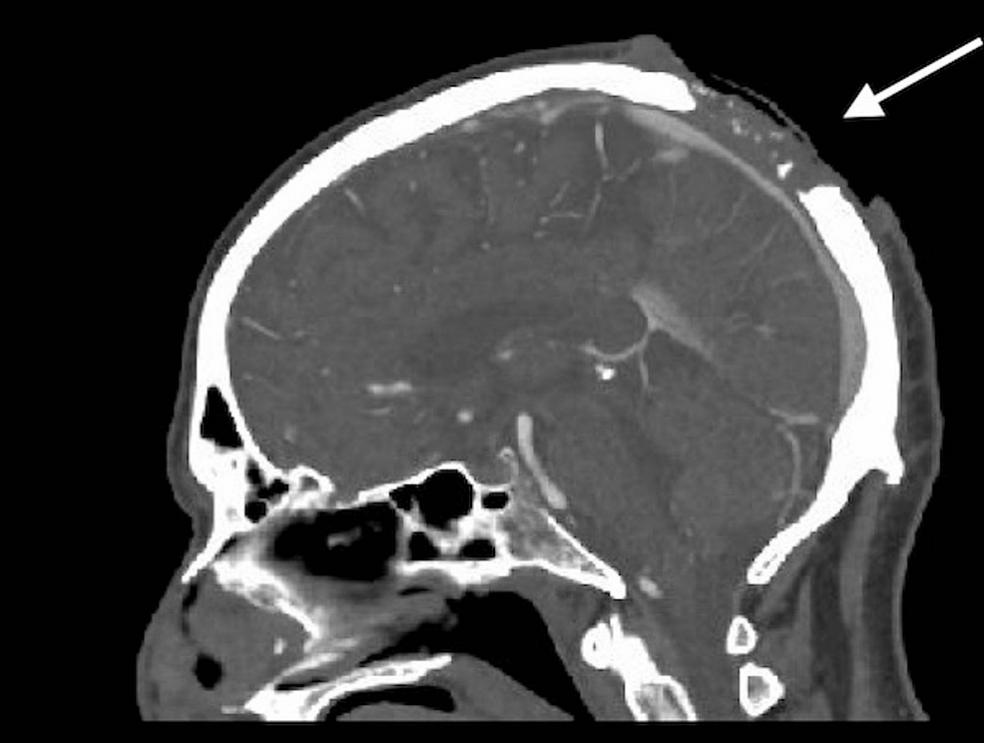
Sagittal images from head CT with contrast demonstrating scalp lesion invading the skull

The case subject was a 65-year-old male who presented with a large ulcerative lesion on his scalp. The lesion appeared three years prior to his presentation; however, the patient did not seek medical evaluation until he had a fall that resulted in a laceration. At the time of his fall, a biopsy was performed and revealed invasive cSCC. Unfortunately, the patient was lost to follow-up and was unable to be notified of his diagnosis. Ten months after this encounter, his family requested a welfare check due to an unexpected lapse in communication, at which point first responders noted the large scalp lesion and brought him to his local emergency department for evaluation. A head CT scan revealed a large scalp lesion invading the skull (Figure 1). He was ultimately transferred to our academic institution for a higher level of specialized care.

**Figure 2:**
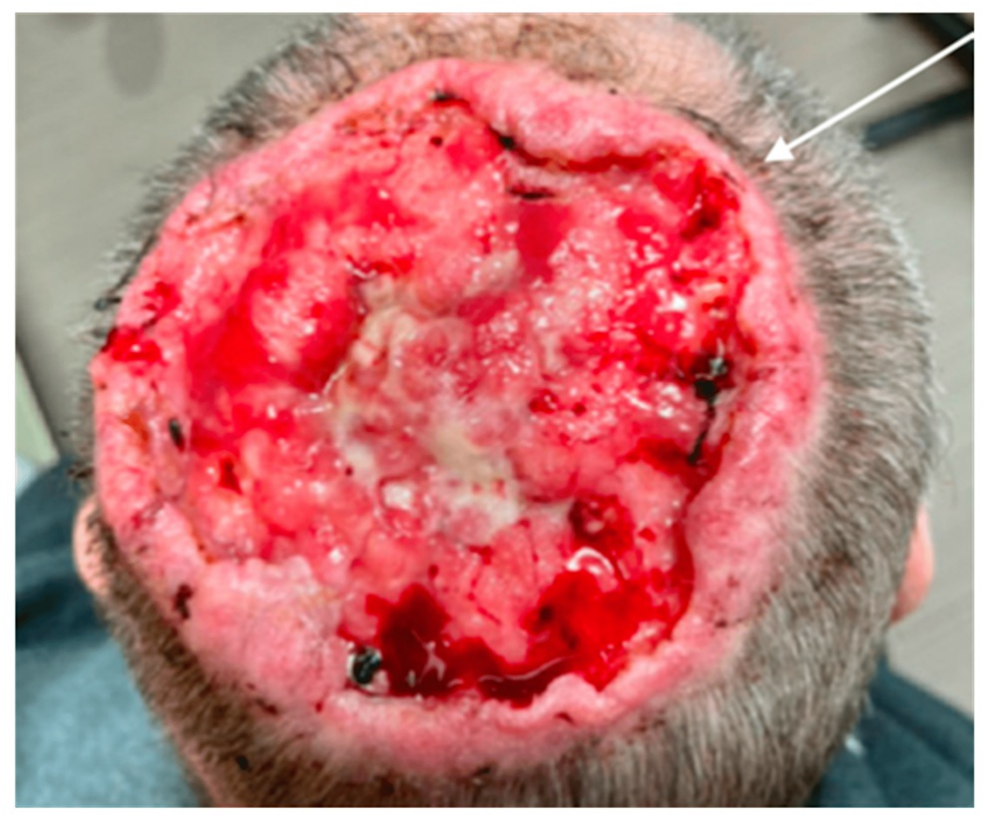
Cutaneous squamous cell carcinoma on the vertex of the scalp measuring 10 cm in diameter

A review of systems was notable for a 10-pound unintentional weight loss over the last year. Other past medical history included tobacco and alcohol use, but he otherwise had no diagnosed comorbid conditions. His physical exam was notable for a 10 cm circumferential, ulcerative, necrotic lesion at the midline vertex of the scalp with rolled edges and a pale complexion (Figure 2). His exam was otherwise unremarkable, including no palpable cervical lymphadenopathy and a normal ear exam. A punch biopsy and a small debridement of the wound edge were performed and resulted in the production of foul-smelling purulent drainage, which was cultured. He was admitted for further evaluation and wound care.

Empiric ampicillin/sulbactam 3000 mg IV every six hours was started and was later narrowed to amoxicillin/clavulanate 875-125 mg tablet every 12 hours after cultures grew *Klebsiella* and *Proteus*. The final pathology of the biopsy confirmed poorly differentiated invasive cSCC. Positron emission tomography (PET) imaging revealed scattered nodes with low-grade metabolic activity in a left 0.9 x 0.5 cm suboccipital node with a maximum standardized uptake value (SUV max) of 2.2 and a left 1.2 x 0.9 cm supraclavicular node with an SUV max of 1.8. There was no evidence of distant metastatic disease. The case was reviewed at our institution’s multidisciplinary head and neck tumor board. This tumor board consists of head and neck surgeons, radiation oncologists, medical oncologists, dentists, radiologists, and pathologists. Given that the tumor was slightly deviated to the left side of the scalp and that imaging did not demonstrate dural involvement, the tumor board recommended wide local excision, craniectomy, left posterolateral and bilateral suboccipital neck dissections, cranioplasty, and reconstruction with a free flap. For reconstruction, a latissimus dorsi free flap was recommended due to the large defect area that would need to be covered. Other flap options for reconstruction were considered but felt to be insufficient for the size of the defect. Neoadjuvant treatment was not recommended in this case as surgery was considered to be curative for this patient and he did not have comorbidities making him a poor surgical candidate.

**Figure 3:**
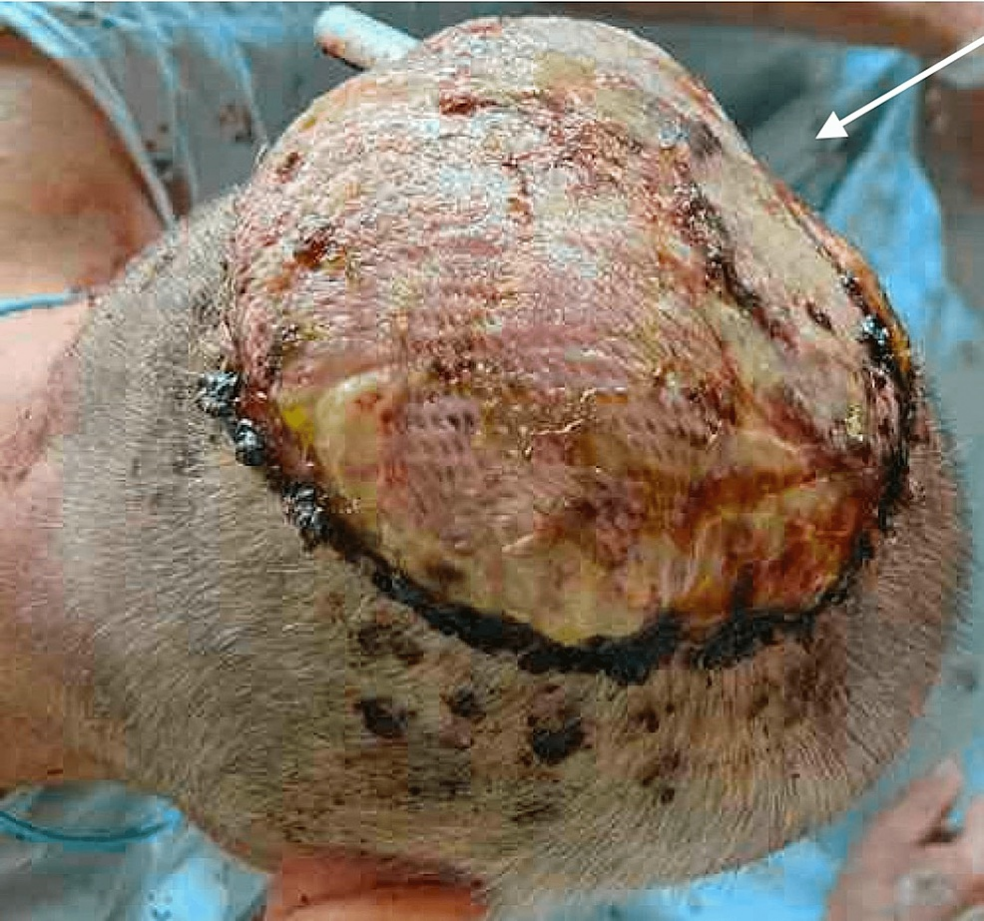
Polyetheretherketone cranioplasty, left latissimus dorsi free flap, and split-thickness skin graft repair of scalp defect

On the day of surgery, the patient underwent a left level II, III, V, and bilateral suboccipital neck dissections, wide local excision of the scalp cSCC, and biparietal craniectomy. Although dural involvement was not previously demonstrated on imaging, during the resection, the dura appeared to be involved with the tumor. The tumor was resected from the dura to the level of the superior sagittal sinus. Further disease resection was determined to carry too high of a risk of mortality and stroke and was thus limited. The final defect measured 13 x 12 cm. He received a polyetheretherketone (PEEK) cranioplasty and left latissimus dorsi myofascial free flap with anastomosis to the superficial temporal artery and vein. A split-thickness skin graft from the left thigh was used to cover the flap (Figure 3). Final pathology demonstrated invasive, poorly differentiated squamous cell carcinoma (SCC), which was 6 cm in the largest dimension, and all peripheral margins were clear of disease; however, the deep margin of the dura overlying the superior sagittal sinus was positive. All lymph nodes included in the neck dissection specimen were negative for SCC. There was no metastatic involvement based on pre-operative CT, MRI, and PET scan findings. Thus, his SCC was staged as stage IV pT4aN0M0.

The patient’s postoperative hospital course was uneventful. His length of stay was 14 days for coordination of discharge needs as he required a skilled nursing facility for wound care. Wound care consisted of routine split-thickness skin graft care with a Xeroform dressing and drain care. After discharge, he received care in a long-term acute care facility for 18 days for rehabilitation and wound management. At his last office visit, he was doing well with expected postoperative changes and adequate healing of the skin graft, free flap, and donor site. Due to the positive deep margin at the dura, the tumor board recommended he receive cisplatin-based adjuvant chemoradiation. Unfortunately, the patient was followed for only two months postoperatively and was again lost to follow-up and attempts to contact him have failed.

## Discussion

cSCC affects 41 of every 100,000 North Americans annually [4], with persistently increasing incidence [1]. cSCC is not only locally invasive but also has metastatic potential and a relatively high recurrence rate [5]. There are several significant risk factors associated with cSCC, including UV light exposure, lower Fitzpatrick skin types, immunosuppression, radiation, chronic wounds, viral infections, and various environmental exposures [3,4,6]. cSCC is also three times more common in men when compared to rates in women [3]. Tobacco use, male sex, and fair complexion are all contributing risk factors seen in this patient.

Given its association with UV exposure, cSCC most commonly affects the head, neck, hands, and forearms [4] with head and neck lesions accounting for 80-90% of cases [2]. In a retrospective study by Derebaşınlıoğlu (2021), it was found that the incidence of cSCC is 11 times higher on the face than on the rest of the body [7]. The presentation of cSCC often consists of a chronic, non-healing wound that may ulcerate or necrose [6]. While overall mortality of cSCC is favorable at 0.7%, metastasis significantly impacts the overall prognosis [1]. The occurrence rate of metastasis is relatively low (0.3-16%) but decreases the overall survival to 34.4% [1]. Modes of metastasis include lymphatic spread, perineural invasion, and direct extension to adjacent tissues [8]. Although cSCC rarely has nodal metastasis, a prospective analysis of lymphatic drainage by Janković et al. found that the anterior scalp more often involves level II notes, while the posterior scalp has a higher incidence of occipital node involvement [1]. Surprisingly, although our patient’s definitive therapy was delayed, he remained free from nodal or distant metastatic disease. Most cSCC are treated with surgery alone; however, radiation can provide additional benefits with certain high-risk features [2,3]. There is some controversy as to when radiation therapy is necessary, but evidence suggests that tumors with positive margins, perineural invasion, higher T stage, high-grade dysplasia, and cervical/parotid nodal involvement may benefit from radiation therapy treatment [2]. Adjuvant chemoradiation was recommended in this case due to the positive dural margin.

Given its high exposure to UV light, the scalp is a site frequently affected by cSCC [9]. cSCC of the scalp has a relatively poor prognosis when compared to other areas of the body due to the vertical growth limitation, high vascularity, ability to develop large diameter lesions before invading critical structures, late detection, and close relation to the brain [5]. The most significant prognostic indicator in cases of cSCC is the depth of invasion [6]. Additionally, involvement of the dura in scalp cSCC decreases three-year survival from 83% to 22% and is often considered inoperable [4,6]. If surgical intervention is a viable option in these cases, collaboration with neurosurgery is critical to ensure a safe and complete resection of the tumor [6]. Overall tumor size larger than 10 mm on the scalp is also associated with a worse prognosis [7]. The delay in definitive care in this case likely contributed to the development of a lesion that was large in diameter with the involvement of deep tissues. These characteristics may have a significant bearing on our patient’s prognosis given his late presentation and involvement of the underlying dura.

Closure of the scalp defect presents a unique challenge as the skin tends to be inelastic and may hold high levels of tension [6,9]. These challenges are further magnified in the elderly, those with poor surrounding tissue, patients with multiple comorbidities and/or a history of prior surgery, or prior radiotherapy affecting the area [4,9]. While small lesions less than 3 cm can likely be repaired primarily with simple undermining, larger defects often need a more complex repair [4,10]. It is important to consider the five tissue layers of the scalp when planning reconstruction, which includes the skin, subcutaneous tissue, galea, which is continuous with temporoparietal fascia laterally and the occipitofrontalis muscle anteroposteriorly, loose areolar tissue, and pericranium [9]. Although the galea is avascular in appearance, it has many small blood vessels running through it that create a strong base for skin grafts [9].

In patients with poor local tissue, large defects, or in need of adjuvant radiation therapy, a latissimus dorsi regional or free flap may be a preferred option for reconstruction [4]. A latissimus dorsi flap is often the preferred method in the scalp as it covers a large surface area and has a long vascular pedicle [10]. A free flap brings a rich blood supply that can improve wound healing, decrease infection risk, and allow the graft to withstand postoperative radiation therapy [4,6,10]. Disadvantages of a free flap approach include loss of hair-bearing skin as well as color and contour mismatch [6]. However, the contour may improve as the muscle atrophies [6,10]. While free flaps are thought to incur a higher risk of reoperation in the immediate postoperative period due to the complexity of the procedure, skin grafting alone is often not durable enough to withstand radiation therapy [6,10]. A retrospective review performed by Eck et al. found that the rate of reoperation was not significantly different from the rate in local flaps or skin grafts. Alternative options for free flap reconstruction include anterolateral thigh and trapezius flaps [6].

These considerations heavily impacted the surgical approach in this case study. Given how locally advanced this tumor was, the patient would need adjuvant radiation therapy. This, in addition to the size of the final defect, made reconstruction with a latissimus dorsi myofascial free flap the preferred option. The involvement of the skull and the dura also posed a reconstructive challenge, necessitating a PEEK cranioplasty. Multidisciplinary collaboration between surgical and medical specialties allowed a complete resection and reconstruction that will withstand future radiation therapy to provide local and regional disease control.

## Conclusions

cSCC is often managed surgically. Scalp involvement with cSCC presents challenging resection and reconstruction. This challenge is further magnified in the case of large, locally advanced cases. These cases require a multidisciplinary collaboration between different surgical and medical teams to provide complete resection of the tumor and prevent or decrease the local and regional risk of recurrence with adjuvant treatment.
